# Host-Derived Leukotriene B_4_ Is Critical for Resistance against Invasive Pulmonary Aspergillosis

**DOI:** 10.3389/fimmu.2017.01984

**Published:** 2018-01-11

**Authors:** Alayna K. Caffrey-Carr, Kimberly M. Hilmer, Caitlin H. Kowalski, Kelly M. Shepardson, Rachel M. Temple, Robert A. Cramer, Joshua J. Obar

**Affiliations:** ^1^Department of Microbiology and Immunology, Montana State University, Bozeman, MT, United States; ^2^Department of Microbiology and Immunology, Geisel School of Medicine at Dartmouth, Lebanon, NH, United States

**Keywords:** *Aspergillus fumigatus*, leukotrienes, leukotriene B_4_, neutrophils, eosinophils, hypoxia inducible factor-1α, respiratory tract infections, fungal infection

## Abstract

*Aspergillus fumigatus* is a mold that causes severe pulmonary infections. Our knowledge of how immune competent hosts maintain control of fungal infections while constantly being exposed to fungi is rapidly emerging. It is known that timely neutrophil recruitment to and activation in the lungs is critical to the host defense against development of invasive pulmonary aspergillosis, but the inflammatory sequelae necessary remains to be fully defined. Here, we show that 5-Lipoxygenase (5-LO) and Leukotriene B_4_ (LTB_4_) are critical for leukocyte recruitment and resistance to pulmonary *A. fumigatus* challenge in a fungal-strain-dependent manner. 5-LO activity was needed in radiosensitive cells for an optimal anti-fungal response and *in vivo* LTB_4_ production was at least partially dependent on myeloid-derived hypoxia inducible factor-1α. Overall, this study reveals a role for host-derived leukotriene synthesis in innate immunity to *A. fumigatus*.

## Introduction

*Aspergillus fumigatus* is a ubiquitous mold that causes severe infections, such as invasive pulmonary aspergillosis (IPA), in the immunocompromised population. Due to a combination of (i) difficulty in diagnosis, (ii) limited efficacy of anti-fungal drugs coupled with the emergence of drug resistance, and (iii) a lack of an effective vaccine against *Aspergillus* spp., mortality rates of IPA are extremely high (1, 2). To this end, development of novel immunomodulatory strategies that can potentially be combined with current anti-fungal treatments is an active area of research.

On a day-to-day basis, inhaled spores are removed from the body through physical barriers encountered within the respiratory tract. If spores are deposited in the lung, resident alveolar macrophages and CCR2^+^ monocytes, together with alveolar epithelial cells, phagocytose, and kill fungal conidia (3, 4). However, in individuals that lack this immune response, conidia are able to germinate and grow within the lung causing tissue damage and disease. These initial encounters are important in the recruitment and activation of neutrophils, inflammatory monocytes, NK cells, and CD4 T cells to further control fungal growth within the lung (5). Of these, neutrophils have long been recognized as one of the key effector cells necessary for resistance against *Aspergillus* infection and neutropenia is a key risk factor for patients that will develop IPA (6).

Neutrophil recruitment and activation is a highly controlled process that is regulated by a number of different inflammatory mediators including C5a, PAF, fMLP, Leukotriene B_4_ (LTB_4_), CXCR2 ligands, CCR1 ligands, TNFα, and IL-17 (7, 8). However, our understanding of the inflammatory mediators driving neutrophil accumulation and activation following *A. fumigatus* challenge remains incomplete. Following *A. fumigatus* challenge, IL-1RI/MyD88 signaling is essential for optimal production of CXCL1 that is necessary for early neutrophil recruitment through CXCR2 (6, 9, 10). In addition, an unknown CARD9-dependent pathway is critical for late neutrophil accumulation following *A. fumigatus* challenge (9). Additionally, a TLR9/Btk/calcineurin/NFAT-dependent pathway regulates neutrophil accumulation during aspergillosis through its regulation of TNFα (11). Moreover, further complexity exists in that *A. fumigatus* isolates with differing virulence depend on distinct inflammatory responses to maintain host resistance (12, 13). Thus, greater knowledge about the inflammatory pathways which contribute to the anti-*Aspergillus* neutrophil response is required.

Regulation of inflammatory responses by lipid mediators is an emerging area. Particularly, LTB_4_ has a critical role in the early recruitment and activation of neutrophils in other inflammatory models (14). Additionally, lipid mediators have been shown to be critical regulators of the host immune response against pathogenic fungi. Following challenge of alveolar macrophages or resident mouse peritoneal macrophages with *Candida albicans* arachidonic acid is effectively mobilized by cPLA2 (15–17), which is necessary for production of both prostaglandins and leukotrienes. cPLA2 is critical for host resistance against *C. albicans* challenge, through the regulation of macrophage transcriptional responses and likely through increased anti-fungal activity of alveolar macrophages (17, 18). Moreover, exogenous LTB_4_ and LTD_4_ can enhance phagocytosis and killing of *C. albicans* by macrophages (19). Finally, LTB_4_-mediated inflammation is critical for host resistance and neutrophil recruitment during *Histoplasma capsulatum* (20, 21), and *Paracoccidioides brasiliensis* (22, 23) infection. Moreover, LTB_4_-mediated inflammation is critical for establishing memory T cells for the prevention of histoplasmosis (24). Thus, we asked whether LTB_4_ was crucial in the neutrophil response following pulmonary *A. fumigatus* challenge. Here, we show that leukotrienes are produced rapidly after *A. fumigatus* challenge by host cells and play a critical role in the anti-fungal neutrophil response necessary for host resistance against pulmonary *A. fumigatus* challenge.

## Materials and Methods

### Mice

C57BL/6J (Stock #000664), C57BL/6NJ (Stock #005304), B6.129S2-*Alox5^tm1Fun^* (*Alox5*^−/−^; Stock #004155), and B6.129S4-*Ltb4r1^tm1Adl^* (*Ltb4r1*^−/−^; Stock #008102) were purchased from Jackson Laboratories. Mice with a targeted deletion of *Hif1a* in myeloid cells were created via crosses into a background of lysozyme M-driven cre-recombinase (*Hif1a*^*LysM/LysM*^), as previously done (30). All mice were 8–10 weeks of age at the time of infection. All animal experiments were approved by the Montana State University Institutional Animal Care and Use Committee or Dartmouth College Institutional Animal Care and Use Committee.

### Preparation of *A. fumigatus* and Pulmonary Challenge Model

*Aspergillus fumigatus* strains CEA10 and Af293 were grown and harvested as previously described (10). For fungal inoculation, mice were anesthetized with isoflurane and challenged by the intratracheally (i.t.) route with 4–7 × 10^7^ *A. fumigatus* conidia in 100 µl sterile PBS. At the indicated time after challenge, mice were euthanized using an overdose of pentobarbital. Samples were collected and analyzed for inflammatory cell recruitment, fungal growth, lung damage, and vascular/epithelial leakage as previously described (10). For survival studies, mice were challenged with 4–7 × 10^7^ conidia of either CEA10 or Af293 and monitored daily using a humane endpoint scoring system. Mice were humanely euthanized once they met endpoint criteria.

### Bone Marrow Chimeric Mice

Bone marrow chimeric mice were made by lethal irradiation of C57BL/6 mice followed by intravenous reconstitution with either C57BL/6 bone marrow or *Alox5*^−/−^ bone marrow. Mice were rested 6–8 weeks prior to challenge with 4 × 10^7^ conidia of CEA10 i.t. At the indicated time-points, mice were euthanized using an overdose of pentobarbital, and samples collected and analyzed for inflammatory cell recruitment and fungal growth as previously described (10).

### Leukotriene Quantification

Lipids were extracted from bronchoalveolar lavage fluid (BALF) using a hot-methanol extraction. Briefly, three parts HPLC-grade methanol were added to one part BALF sample. Samples were then vortexed for 30 s and placed into an 80°C water bath for 2 min. Tubes were spun at 14,000 RPM for 15 min and supernatant was collected then dried using a vacuum concentrator. Pellets were resuspended in HPLC-grade water in a volume equal to the starting volume of BALF sample. Extracted samples were then analyzed using enzyme immunoassay kits for LTB_4_, cysteinyl leukotrienes (cysLT) (Cayman Chemical). Plates were read using a SpectraMax^®^ Paradigm^®^ plate reader (Molecular Devices).

### Statistical Analysis

Statistical significance between experimental groups was determined using a Mann–Whitney *U* test (comparison of two experimental groups that are not normally distributed) or an one-way ANOVA with a Dunn’s post-test (comparison of greater than two experimental groups that are not normally distributed), using the GraphPad Prism 6 software. For survival studies, Mantel–Cox log-rank test was used to determine whether there were significant differences in survival between C57BL/6, Hif1a^LysM/LysM^, *Ltb4r1*^−/−^, and *Alox5*^−/−^ mice for each *A. fumigatus* strain.

## Results

### Leukotriene Production following Pulmonary Challenge with *A. fumigatus*

**Figure 1:**
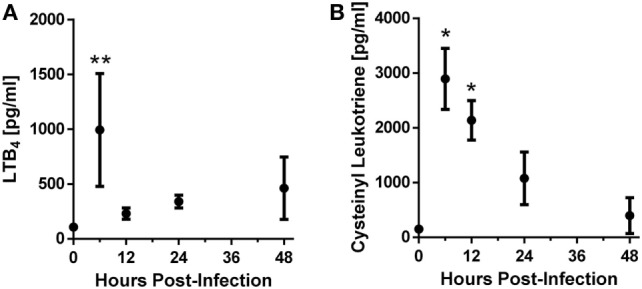
C57BL/6 mice produce leukotrienes after pulmonary challenge with the CEA10 isolate of *Aspergillus fumigatus*. Mice were infected intratracheally with 5 × 10^7^ CEA10 conidia and at indicated time-points, mice were euthanized and bronchoalveolar lavage fluid (BALF) collected. Lipids were then extracted from BALF using a hot-methanol extraction procedure, and LTB4 **(A)** and cysteinyl leukotriene **(B)** levels in the extracted BALF samples were measured using Cayman Chemical enzyme immunoassay kits. Data are representative of five mice per time-point. Each dot represents the mean ± 1 SEM. Statistically significant differences were determined using an one-way ANOVA with a Dunn’s post-test (*p < 0.05 and **p < 0.01).

In order to determine whether leukotrienes are produced following *A. fumigatus* challenge, we challenged C57BL/6 wild-type mice with the CEA10 strain of *A. fumigatus*. Throughout a time course of 6–48 h post-infection (hpi), we collected BALF and measured leukotriene production. We found that LTB_4_ and cysLT showed increased production after *A. fumigatus* CEA10 challenge. The production of these inflammatory lipid mediators followed a similar trend early after infection in which their expression peaked at 6 hpi, followed by decreased levels at 12 hpi. At 24 and 48 hpi LTB_4_ increased from the 12 hpi levels, while the cysLT levels continue to decrease (Figure 1). These data demonstrate that both LTB_4_ and cysLTs are synthesized following challenge of immunocompetent mice with *A. fumigatus*.

### *Ltb4r1*^−/−^ Mice Have a Defect in Inflammatory Cell Recruitment and Resistance to IPA

**Figure 2:**
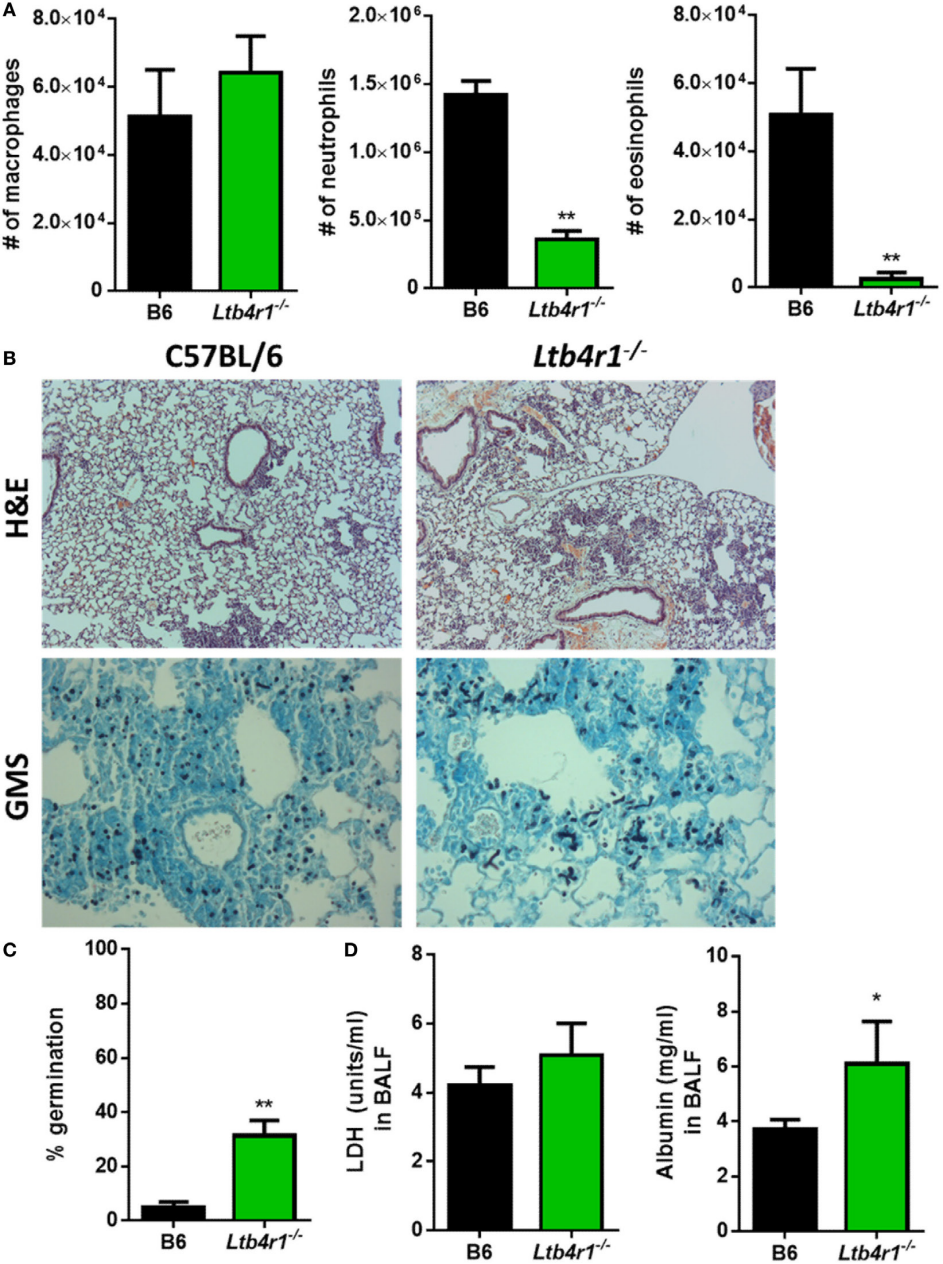
*Ltb4r1*-deficient mice have a defect in neutrophil and eosinophil recruitment and increased fungal burden after pulmonary *Aspergillus fumigatus* challenge. Age-matched C57BL/6 or *Ltb4r1*-deficient mice were infected intratracheally with 5 × 10^7^ CEA10 conidia and at indicated time-points, mice were euthanized, bronchoalveolar lavage fluid (BALF) collected, and lungs saved for histological analysis. **(A)** Total macrophage (left panel), neutrophil (middle panel), and eosinophil (right panel) numbers in the BALF was measured at 12 h post-infection (hpi). **(B)** Formalin-fixed lungs were paraffin embedded, sectioned, and stained with hematoxylin and eosin (H&E) (top) or Grocott-Gomori methenamine silver (GMS) (bottom) for analysis by microscopy. Representative lung sections from C57BL/6 and *Ltb4r1*-deficient mice infected with CEA10 are shown using the 10× objective for H&E staining or 40× objective for GMS staining. **(C)** *A. fumigatus* germination rates were assessed at 24 hpi by microscopically counting the number of conidia and germlings in GMS-stained sections from C57BL/6 and *Ltb4r1-*deficient mice. **(D)** Lung damage (left panel) and leakage (right panel) were assessed at 24 hpi by measuring lactate dehydrogenase (LDH) and albumin levels in the BALF, respectively. Data are representative of at least two independent experiments consisting of five to eight mice per group. Bar graphs show the group mean ± 1 SEM. Statistically significant differences were determined using Mann–Whitney *U* test (*p < 0.05, **p < 0.01).

LTB_4_ is known to be important in the recruitment of neutrophils in numerous inflammatory settings (14), but whether it is crucial in regulating the innate immune response following *A. fumigatus* challenge is unknown. To address whether LTB_4_ was critical for neutrophil recruitment and resistance against IPA, we challenged *Ltb4r1*^−/−^ and C57BL/6 mice with *A. fumigatus*. At 12 hpi, we analyzed BALF *via* differential cytospins stained with Diff-Quik™ to assess early inflammatory cell recruitment to the airways. Compared with the C57BL/6 mice, *Ltb4r1*^−/−^ mice had a significant defect in neutrophil and eosinophil numbers, while macrophage numbers was similar (Figure 2A). Because the early recruitment of neutrophils is needed for host resistance to invasive *A. fumigatus* infection (6), we next addressed whether fungal growth was enhanced in the *Ltb4r1*^−/−^ animals. At 24 hpi, Grocott-Gomori methenamine silver (GMS) staining of lung sections revealed the presence of an increased proportion of germinated *A. fumigatus* in *Ltb4r1*^−/−^ mice compared with C57BL/6 mice (Figure 2B) demonstrating *Ltb4r1*^−/−^ mice were impaired in their ability to clear the fungi (Figure 2C). Lastly, lung damage and endothelial/epithelial leakage induced by *A. fumigatus* challenge were assessed by quantifying lactate dehydrogenase (LDH) and albumin in the BALF, respectively. Albumin levels were significantly elevated in *Ltb4r1*^−/−^ mice compared with C57BL/6 mice, indicating an increase in protein leakage from the vascular system (Figure 2D). In contrast, LDH levels were only mildly elevated suggesting induction of similar degrees of cell damage (Figure 2D). Taken together, these data demonstrate that LTB_4_ signaling through its high-affinity receptor LTB4R1 is important in mediating neutrophil and eosinophil recruitment to the airways, which was ultimately necessary for host resistance to *A. fumigatus* growth.

### *Alox5*^−/−^ Mice Are Impaired in Inflammatory Cell Recruitment and Resistance to IPA

**Figure 3:**
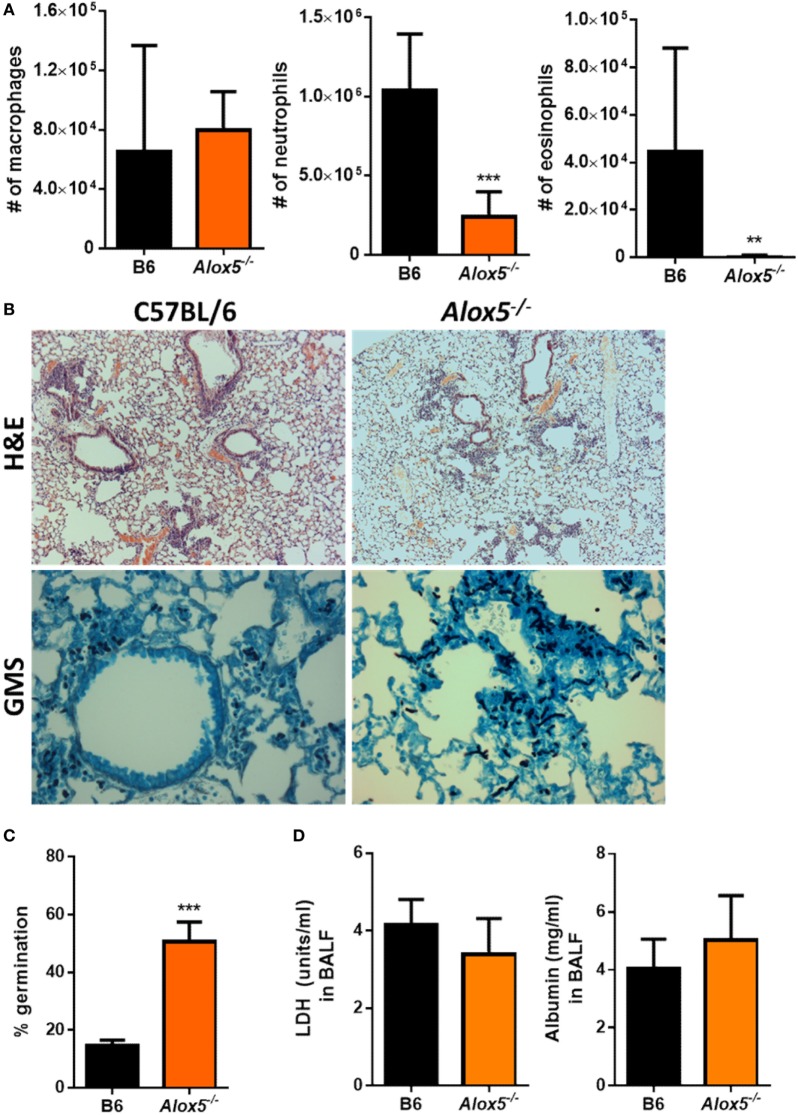
*Alox5*-deficient mice have a defect in neutrophil and eosinophil recruitment and increased fungal burden after pulmonary *Aspergillus fumigatus* challenge. Age-matched C57BL/6J or *Alox5*-deficient mice were infected intratracheally with 5 × 10^7^ CEA10 conidia and at indicated time-points, mice were euthanized [bronchoalveolar lavage fluid (BALF)] collected, and lungs saved for histological analysis. **(A)** Total macrophage (left panel), neutrophil (middle panel), and eosinophil (right panel) numbers in the BALF was measured at 12 hpi. **(B)** Formalin-fixed lungs were paraffin embedded, sectioned, and stained with hematoxylin and eosin (H&E) (top) or Grocott-Gomori methenamine silver (GMS) (bottom) for analysis by microscopy. Representative lung sections from C57BL/6J and *Alox5*-deficient mice infected with CEA10 are shown using the 10× objective for H&E staining or 40× objective for GMS staining. **(C)** *A. fumigatus* germination rates were assessed at 36 hpi by microscopically counting the number of conidia and germlings in GMS-stained sections from C57BL/6J and *Alox5-*deficient mice. **(D)** Lung damage (left panel) and leakage (right panel) were assessed at 36 hpi by measuring lactate dehydrogenase (LDH) and albumin levels in the BALF, respectively. Data are representative of at least two independent experiments consisting of five to eight mice per group. Bar graphs show the group mean ± 1 SEM. Statistically significant differences were determined using Mann–Whitney *U* test (**p < 0.01 and ***p < 0.001).

*Aspergillus fumigatus* itself is known to be capable of producing eicosanoids (25), which results in an infection system in which both the mammalian and fungal cells could be the source of bioactive LTB_4_. Thus, to address whether LTB_4_ production coming from the murine cells was necessary for host resistance against *A. fumigatus*, we challenged 5-lipoxygenase (5-LO) (*Alox5*^−/−^) deficient mice with *A. fumigatus. Alox5*^−/−^ mice cannot convert arachidonic acid to LTA_4_ and, therefore, lack all leukotriene synthesis (22). After *A. fumigatus* challenge, inflammatory cell recruitment to the airways was quantified at 12 hpi *via* cytospins and Diff-Quik™ staining. Similar to what we found with the *Ltb4r1*^−/−^ mice, *Alox5*^−/−^ mice had a significant defect in both neutrophil and eosinophil recruitment, while macrophage accumulation remained largely similar to C57BL/6 (Figure 3A). This defect in neutrophil and eosinophil recruitment correlated with an impairment in the ability of *Alox5*^−/−^ mice to control fungal growth within the lung, demonstrated by a significantly higher germination rate (Figures 3B,C). We also measured LDH and albumin levels in the BALF of the *Alox5*^−/−^ mice to assess lung damage and vascular/epithelial permeability, respectively. Interestingly, unlike the *Ltb4r1*^−/−^ mice, LDH and albumin levels in BALF from *Alox5*^−/−^ mice were not significantly different than C57BL/6 levels (Figure 3D). Together, these data indicate that leukotriene synthesis by host cells is critical for neutrophil and eosinophil recruitment, as well as host resistance against *A. fumigatus* growth.

### Radiosensitive Cells Contribute to 5-LO Activity following Pulmonary *A. fumigatus* Challenge

**Figure 4:**
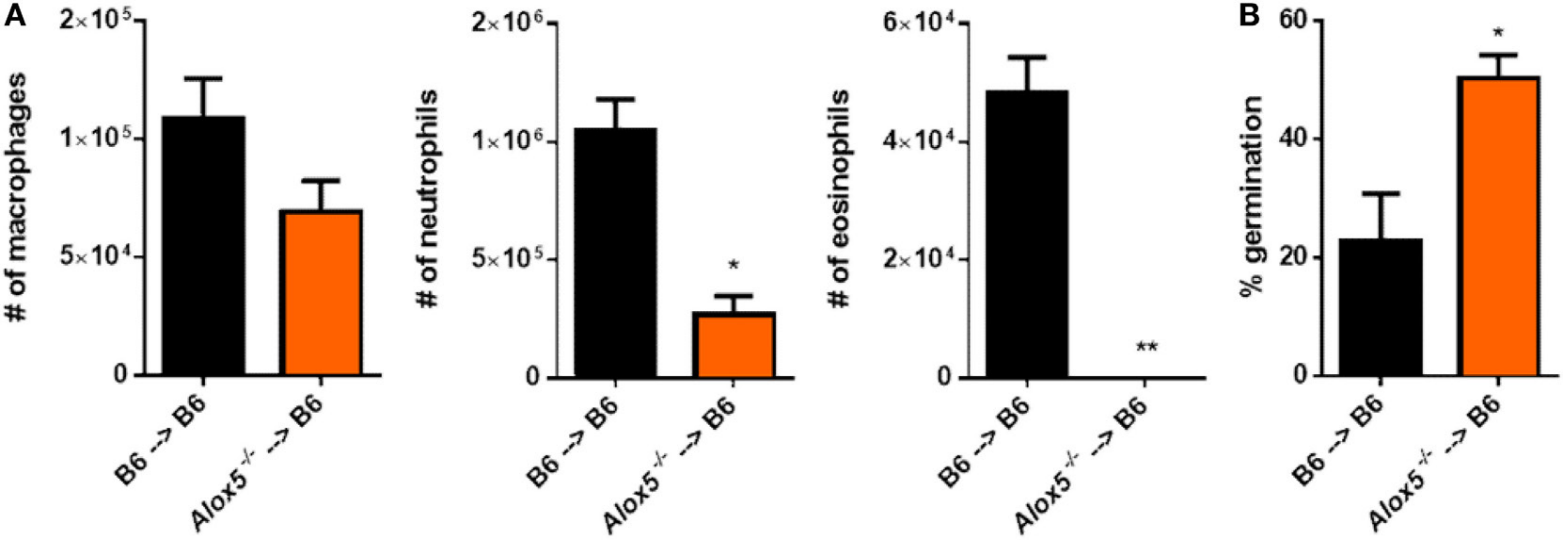
Radiosensitive cells contribute to 5-lipoxygenase activity after pulmonary *Aspergillus fumigatus* challenge. Bone marrow chimeric mice were made by lethally irradiating C57BL/6 mice and reconstituting the mice with bone marrow from either C57BL/6 or *Alox5*^−/−^ mice. These mice were challenged intratracheally with 4 × 10^7^ conidia of CEA10 and at 36 hpi, mice were euthanized and **(A)** bronchoalveolar lavage fluid collected for quantification of leukocyte numbers to the airways and **(B)** lungs saved for histological analysis in which formalin-fixed lung were paraffin embedded, sectioned, and stained with Grocott-Gomori methenamine silver for analysis by microscopy. Data consist of four to five mice per group. Bar graphs show the group mean ± 1 SEM. Statistical significance was determined using a Mann–Whitney *U* test (*p < 0.05 and **p < 0.01).

To begin to determine the cells that contribute to 5-LO activity after *A. fumigatus* challenge, we utilized a bone marrow chimera approach. C57BL/6 mice were lethally irradiated then reconstituted with either C57BL/6 or *Alox5*^−/−^ bone marrow intravenously to develop the following groups: C57BL/6 mice possessing C57BL/6 bone marrow and C57BL/6 mice possessing *Alox5*^−/−^ bone marrow. Mice were then rested for 6–8 weeks prior to challenge with 4 × 10^7^ conidia of CEA10. At 36 hpi, mice were euthanized, BAL collected for analysis of leukocyte recruitment to the airways, and lungs saved for histological analysis to assess fungal growth by GMS staining. Compared with C57BL/6 mice possessing C57BL/6 bone marrow, C57BL/6 mice possessing *Alox5*^−/−^ bone marrow had a significant defect in neutrophil and eosinophil recruitment to the airways at 36 hpi, while macrophage accumulation was not significantly altered (Figure 4A). Moreover, at 36 hpi, C57BL/6 mice possessing *Alox5*^−/−^ bone marrow were impaired in their ability to control fungal germination compared with C57BL/6 mice possessing C57BL/6 bone marrow (Figure 4B). Taken together, these data suggest that radiosensitive cells contribute to 5-LO activity which is needed for neutrophil and eosinophil recruitment to the airways and control of fungal germination following pulmonary *A. fumigatus* challenge.

### Leukotriene Synthesis Is Critical for Host Survival following Challenge with the CEA10 Strain of *A. fumigatus*, but Not the Af293 Strain

**Figure 5:**
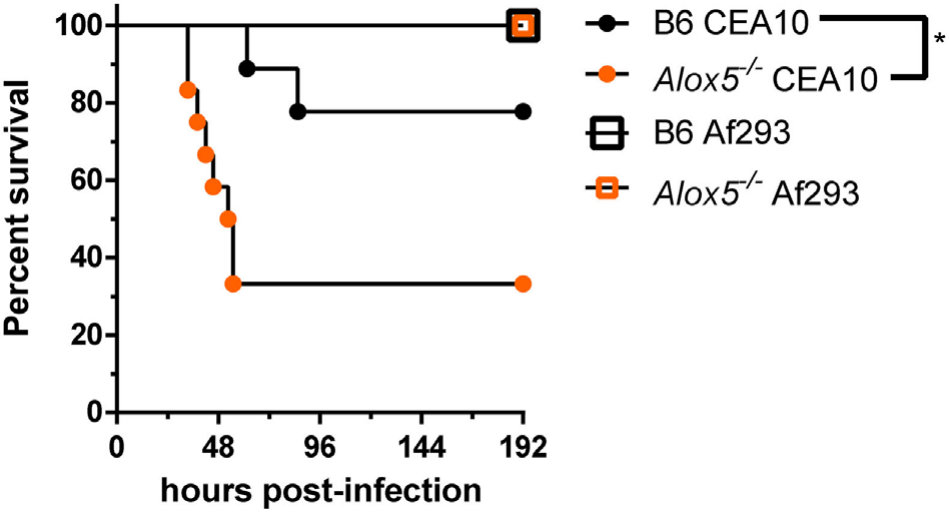
Leukotriene-mediated immunity is fungal-strain dependent. C57BL/6 and *Alox5*-deficient were challenged with 5 × 10^7^ conidia of CEA10 or Af293 intratracheal. Mice were monitored daily using a humane endpoint scoring system (based on weight loss, inactivity, ruffled fur, difficulty breathing, and neurological symptoms), and when the humane endpoint criteria was reached mice were humanely euthanized. Data are representative of 9–12 mice per group. Survival was plotted on Kaplan–Meier curves, and statistical significance between curves determined using the Mantel–Cox log rank (ns, not significant, *p < 0.05).

Thus far, we have shown that in response to the CEA10 strain of *A. fumigatus*, host-derived leukotriene synthesis is critical for timely neutrophil recruitment and control of fungal germination within the lung. Due to studies that demonstrate fungal-strain dependency on the host immune response, we next sought to determine if leukotriene-dependent immunity was reliant on the strain of *A. fumigatus* used for challenge (12, 13, 26). Inflammatory responses against the hypervirulent CEA10 isolate are highly inflammatory and heavily dependent on IL-1α for maintaining host resistance (12). Additionally, we showed that IL-1α release from macrophages following CEA10 challenge required calpain, which is a Ca^2+^-dependent protease. Since, cPLA2 release of arachidonic acid is also a Ca^2+^-dependent event, we hypothesized that strain-specific LTB_4_ responses might be likely. To test this, we challenged C57BL/6 and *Alox5*^−/−^ mice intratracheally with 5 × 10^7^ conidia of either CEA10 or Af293. We monitored health and survival of mice in the 192 h following fungal challenge. *Alox5* (5-LO) was dispensable for survival of mice challenged with the Af293 strain of *A. fumigatus*, which is demonstrated by 100% survival of all mice challenged with Af293 (Figure 5). Conversely, *Alox5*^−/−^ mice challenged with CEA10 displayed significantly higher mortality than C57BL/6 mice. Similar mortality trends were observed in the *Ltb4r1*^−/−^ mice (data not shown). Overall, these data are in agreement with several other studies demonstrating that innate immune responses necessary for host resistance against *A. fumigatus* are highly dependent on the fungal strains used.

### *Hif1a*-Deficient Mice Show a Defect in LTB4 Production following Challenge with CEA10, but Not with Af293

**Figure 6:**
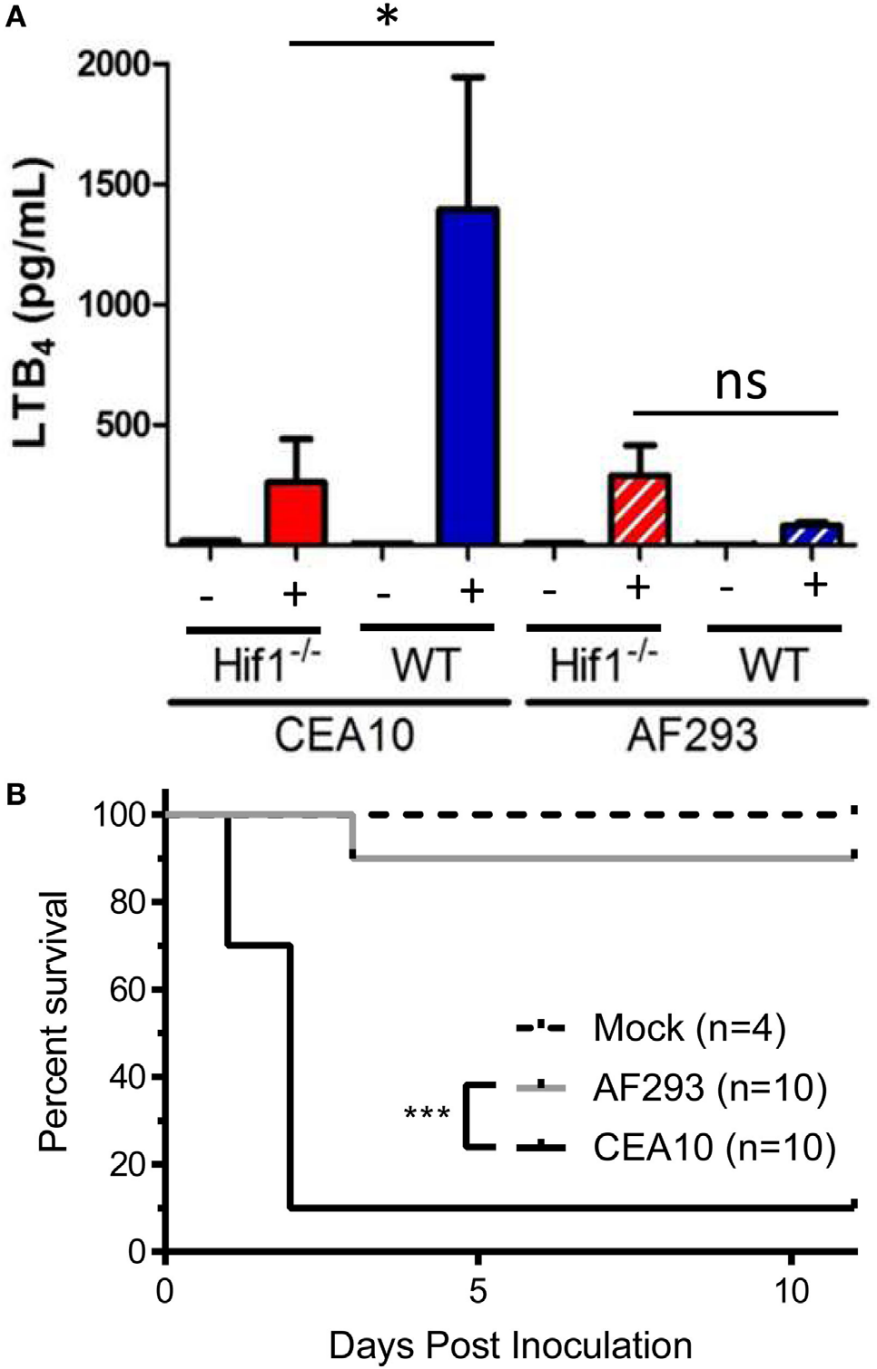
LTB_4_ production following *Aspergillus fumigatus* challenge is dependent on hypoxia inducible factor-1α (HIF-1α). **(A)** C57BL/6 or myeloid-specific lysozyme-M cre-recombinase driven HIF-1α null mice (Hif1^LysM/LysM^) were challenged with either 5 × 10^7^ CEA10 or Af293 by the intratracheal (i.t.) route. At 8 hpi, mice were euthanized, bronchoalveolar lavage fluid collected and LTB_4_ measured. Data consist of four mice per group for *A. fumigatus*-challenged mice and one mouse per group challenged with PBS. Statistically significant differences were determined by a one-way ANOVA with a Dunn’s post-test (ns, not significant, *p < 0.05). **(B)** C57BL/6 or myeloid-specific lysozyme-M cre-recombinase driven HIF-1α null mice (Hif1^LysM/LysM^) were challenged with either 7 × 10^7^ CEA10 or Af293 by the i.t. route. Mice were monitored daily using a humane endpoint scoring system (based on weight loss, inactivity, ruffled fur, difficulty breathing, and neurological symptoms), and when the humane endpoint criteria was reached mice were humanely euthanized. Data are representative of 10 mice per experimental group or 4 mice in the uninfected control group. Survival was plotted on Kaplan–Meier curves, and statistical significance between curves determined using the Mantel–Cox log rank (***p < 0.0001).

Under hypoxic conditions, the transcription factor hypoxia inducible factor-1α (HIF-1α) is activated, which allows translocation to the nucleus where it can bind to hypoxia response elements in the promoter region of target genes (27). HIF-1α is involved in controlling expression of 5-LO activating protein, a protein critical for the biosynthesis of LTB_4_ and cysLTs, after hypoxic challenge (28). Previous work has shown that hypoxic microenvironments form within the lung in multiple murine models of IPA (29). In an immune competent model of fungal bronchopneumonia, myeloid-derived HIF-1α was shown to be critical for neutrophil recruitment and ultimately, survival of mice challenged with *A. fumigatus* CEA10 strain (30). Given the link between HIF-1α and leukotriene biosynthesis, we sought to determine if HIF-1α contributes to LTB_4_ production following *A. fumigatus* challenge. To test this, C57BL/6 or myeloid-specific lysozyme-M cre-recombinase driven HIF-1α null mice (*Hif1a^LysM/LysM^*) mice were challenged with 5 × 10^7^ conidia of either CEA10 or Af293, and LTB_4_ protein levels measured in the BALF at 8 hpi. Following challenge with CEA10, there was a significant decrease in LTB_4_ protein released into the airways of *Hif1a^LysM/LysM^* mice compared with C57BL/6 mice (Figure 6A). Interestingly, following Af293 challenge LTB_4_ levels remained unchanged between C57BL/6 and *Hif1a^LysM/LysM^* mice (Figure 6A). Overall, this suggests that HIF-1α contributes to control of LTB_4_ release following pulmonary *A. fumigatus* challenge in a fungal-strain-dependent manner.

Due to strain-specific release of LTB_4_ in *Hif1a^LysM/LysM^* mice (Figure 6A), we next sought to determine if *Hif1a^LysM/LysM^* mice displayed a strain-specific susceptibility to *A. fumigatus* challenge. To test this, we challenged C57BL/6 and *Hif1a^LysM/LysM^* mice intratracheally with 7 × 10^7^ conidia of either CEA10 or Af293. We monitored health and survival of mice in the 11 days following fungal challenge. HIF-1α expression in LysM-expressing cells was dispensable for survival of mice challenged with the Af293 strain of *A. fumigatus*, which is demonstrated by 90% survival of all mice challenged with Af293 (Figure 6B). Conversely, *Hif1a^LysM/LysM^* mice challenged with CEA10 displayed significantly higher mortality (10% survival), as previously reported (Figure 6B). Overall, these data are in agreement with the survival analysis of the *Alox5*^−/−^ mice and reiterate that the innate immune responses necessary for host resistance against highly virulent *A. fumigatus* isolates, such as CEA10, are highly dependent on a robust inflammatory response.

## Discussion

This study reveals an essential role for host-derived LTB_4_ in the recruitment of both neutrophils and eosinophils through its high-affinity chemotactic receptor LTB4R1 following pulmonary challenge with high-virulent *A. fumigatus* strain CEA10. Ultimately, 5-LO and LTB_4_ were crucial for host resistance to *A. fumigatus* challenge. In addition to recruitment of neutrophils, it was recently shown that LTB_4_-treated neutrophils have enhanced anti-fungal activity against *A. fumigatus* (31). Thus, LTB_4_ will not only recruit neutrophils to the site of *A. fumigatus* infection, but also simultaneously activates their anti-fungal armory. While our data are the first to demonstrate a critical role for LTB_4_ in regulating neutrophil recruitment following challenge with a mold, others have reported leukotrienes are critical for leukocyte recruitment and activation following infection with the dimorphic fungi, *H. capsulatum* and *P. brasiliensis* (20, 22, 32). In these systems, it is unknown how leukotriene synthesis is initiated, but with the yeast *C. albicans* treatment of macrophages *in vitro* results in the synthesis of leukotrienes through a Dectin1-, Dectin2-, and MyD88-dependent pathway (16). Given that Dectin1, Dectin2, and MyD88 play a role in recognizing not only *A. fumigatus* (9, 10, 33, 34), but a range of fungal pathogens, it is likely this pathway will be important in the induction of the inflammatory eicosanoid pathway universally following challenge with fungal pathogens.

While it is well known that neutrophils are one of the most important effector cells against *A. fumigatus* (5), a recent study demonstrated a potential role for eosinophils in limiting the development of IPA (35). Our data demonstrate that LTB_4_ is also critical for the recruitment of eosinophils to the airways of *A. fumigatus*-challenged mice. In immunocompetent mice, the absence of eosinophils resulted in a defect in *A. fumigatus* clearance *in vivo* which did not correlate with a defect in recruitment or function of other inflammatory cells, but rather the potential direct anti-fungal activity of eosinophils (35). In contrast, with repeated administration of *A. fumigatus* eosinophils were shown to have a detrimental effect on disease outcome and eosinophilia was associated with a decrease in neutrophil recruitment (36). Thus, more studies are needed to determine the exact role eosinophils play in different experimental models of IPA, and whether crosstalk between eosinophils and neutrophils regulate anti-fungal immunity.

Clinically, patients with chronic granulomatous disease have a higher risk of developing invasive *Aspergillus* infections, which is mirrored in mice lacking the NADPH oxidases. In these mice, IPA is characterized by excessive inflammation and tissue damage (37, 38). Interestingly, when *gp91^phox^*-deficient mice were exposed subcutaneously to *A. fumigatus* there is an early neutrophilic response which is associated with elevated LTB_4_ levels (38). Thus, it will be intriguing whether limiting LTB_4_ signaling can ameliorate IPA in *gp91^phox^*-deficient mice, similarly to what was observed with anakinra treatment to limit IL-1 signaling (37). This is plausible because in a *Mycobacterium tuberculosis* model, eicosanoid and IL-1 signaling regulate *M. tuberculosis* growth and pathogenicity (39). Furthermore, LTB_4_ antagonism is an attractive treatment, because there are a number of clinically available inhibitors of this pathway. Another patient population at high risk of developing IPA is individuals receiving long-term corticosteroid treatment. Treatment with glucocorticoids leads to inhibition of cytosolic phospholipase A2α through the induction of lipocortin-1, blocking the conversion of phospholipids to arachidonic acid (40). Given this link between glucocorticoid treatment and the arachidonic acid pathway, it would be interesting to see the overall impact the presence or absence of leukotrienes has on the outcome of disease during a corticosteroid model of IPA. Moreover, in mice challenged with *A. fumigatus* corticosteroid treatment inhibited HIF-1α translocation to the nucleus, where it binds to target genes to regulate transcription (30). Due to the HIF-1α-dependent production of LTB_4_ (Figure 5), it will be important to determine if the impaired HIF-1α translocation to the nucleus contributes to impaired LTB4 production, which results in enhanced susceptibility to IPA in those patients. Finally, these data suggest that the susceptibility of *Hif1*α-deficient mice to IPA may also be fungal-strain dependent, which warrants further dissection.

In conclusion, we have shown that during the inflammatory response of immunocompetent mice following pulmonary challenge with *A. fumigatus*, leukotriene production from hematopoietic cells is induced. In the absence of all leukotriene synthesis, and specifically in the absence of LTB4R1 signaling, there is a significant defect in neutrophil and eosinophil recruitment to the airways. This defect in leukocyte recruitment correlated with an elevated susceptibility of these mice to developing IPA, as measured by enhanced fungal germination in the lungs and enhanced mortality of mice devoid of leukotriene synthesis. Furthermore, the enhanced susceptibility of mice lacking leukotriene synthesis was fungal-strain dependent. Overall, these data reveal that the leukotriene pathway is critical in maintaining host resistance against *A. fumigatus* infection. Interestingly, prostaglandin E2 was recently shown to be involved in the anti*-Aspergillus* immune response (41), demonstrating a generally important role for arachidonic acid metabolites in regulating anti-fungal immunity.

## Ethics Statement

All animal experiments were approved by the Montana State University Institutional Animal Care and Use Committee or Dartmouth College Institutional Animal Care and Use Committee.

## Author Contributions

AC-C, CK, KS, RC, and JO conceived and designed the experiments. AC-C, KH, CK, KS and RT performed the experiments. AC-C, KH, CK, KS, RT, RC, and JO analyzed the data. AC-C and JO wrote the paper. AC-C, RC, and JO edited the paper.

## Conflict of Interest Statement

The authors declare that the research was conducted in the absence of any commercial or financial relationships that could be construed as a potential conflict of interest.
